# Non communicable diseases persist despite being overshadowed by Covid-19 globally

**DOI:** 10.4314/ahs.v21i1.1

**Published:** 2021-03

**Authors:** James K Tumwine

**Affiliations:** Editor in Chief, African Health Sciences. kabaleimc@gmail.com

In this March 2021 issue, we start with interesting papers on non-communicable diseases.

Thus, Jian Ma and colleagues have a meta-analysis on the efficacy of dapagliflozin in type 1 diabetes mellitus.^1^ Keeping with the diabetes theme we have papers on vitamin D receptor gene in patients with type 2 diabetes mellitus,^2^ TCF7L2 gene polymorphism^3^, overweight^4^ and knowledge, attitude and practice in the Sudan^5^. Continuing with the NCD theme, we bring you papers on cancer of the breast^6^,^7^, childhood tumors^8^, oncology nursing care in Mexico^9^, and multiple myeloma in Uganda.^10^ We then have papers on hypertension^11^,^12^ cholesterol and aortic dilatation^13^, and heart rates in smoker and nonsmoker athletes.^14^ You talk of smoking! Gazibara and others write for us an interesting article on differences among former light and heavy smokers in a post conflict setting.^15^ Then comes the paper on risk factors for hazardous drinking among South African and Belgian University Students.^16^

Nyoni-Kachambwa's paper on skin bleaching^17^ in Zimbabwe is a must read and so is Celik's on p wave dispersion associated with obstructive pulmonary disease.^18^

Papers on sickle cell anemia^19^, IgA nephropathy^20^, and pseudobulbar palsy^21^, spice this series.

Olusegun-Joseph et. al have written on mortality in an emergency department in Nigeria^22^, while Sarwar's paper^23^ on MAO-A gene association with aggression in Pakistan concludes this NCD section.

## Infections

Now to infections. Yes, you guessed right! We have failed to resist the temptation and included papers on Covid-19: roles of exercise and immunity 24, and mortality among Indian patients;^25^ and a paper on Corona-Virus reaching far beyond the common cold.^26^ So we are deep in the infectious disease territory: risk factors for multiple drug resistant bacteria in urinary tract infections (UTI)^27^, and UTI in nurses and civil servants.^28^

We have several papers on TB: hearing-threshold in drug-resistant TB^29^; non adherence to MDR-TB treatment in Uganda^30^, and TB/HIV service integration in Kampala.^31^ HPV/HIV confection in Kenya^32^, and first line HIV treatment failure in Ethiopia^33^, conclude the HIV papers. We have a series of papers on malaria^34^, septic shock 35, and wound infection with MRSA^36^ concluding the infections disease section.

## Reproductive and child health

Now to reproductive and child health. There are papers on ectopic pregnancy in the Gambia^37^, postpartum haemorrhage management in Kenya^38^ and South Africa^39^ and Caesarean section deliveries in Ibadan in Nigeria. 40 We also have papers on married women procuring abortions^41^, fetal ear length 42, infertility 43, and paternity in motherless children.^44^ These are followed by papers on birth length^45^, neonatal asphyxia^46^, and neonatal mortality^47^, and factors associated with underweight in Ethiopia.^48^

Then we have a cocktail of papers on anti-inflammatory potential of *Eucalyptus*^49^, herbal medicine use in Ethiopia^50^, and traditional bone-setting in Tanzania^51^.

This ushers in *surgical* issues: intestinal stoma in Uganda^52^, phthisis bulbi in Nigeria 53, cataract surgery in Ghana 54, mercury in dentstry^55^, dental health insurance hygiene^56^, and ethical issues in seeking consent for health procedures in the Democratic Republic of the Congo.^57^ We hope that you will enjoy this hybrid issue of AHS. Good reading.
